# Target Cell Extraction and Spectrum–Effect Relationship Coupled with BP Neural Network Classification for Screening Potential Bioactive Components in Ginseng Extract with a Protective Effect against Myocardial Damage

**DOI:** 10.3390/molecules29092028

**Published:** 2024-04-28

**Authors:** Junyi Li, Min Lin, Zexin Xie, Liwenyu Chen, Jin Qi, Boyang Yu

**Affiliations:** 1 Research Center for Traceability and Standardization of TCMs, School of Traditional Chinese Pharmacy, China Pharmaceutical University, Nanjing 211198, China; lijunyi0110@163.com (J.L.); 18355092310@163.com (M.L.); xzx622425@163.com (Z.X.); clwy1997@163.com (L.C.); 2 Jiangsu Key Laboratory of TCM Evaluation and Translational Research, School of Traditional Chinese Pharmacy, China Pharmaceutical University, Nanjing 211198, China

**Keywords:** red ginseng, gray relationship analysis: partial least squares discrimination analysis, target cell extraction, bioactive compounds, BP neural network classification

## Abstract

Cardiovascular disease has become a common ailment that endangers human health, having garnered widespread attention due to its high prevalence, recurrence rate, and sudden death risk. Ginseng possesses functions such as invigorating vital energy, enhancing vein recovery, promoting body fluid and blood nourishment, calming the nerves, and improving cognitive function. It is widely utilized in the treatment of various heart conditions, including palpitations, chest pain, heart failure, and other ailments. Although numerous research reports have investigated the cardiovascular activity of single ginsenoside, there remains a lack of systematic research on the specific components group that predominantly contribute to cardiovascular efficacy in ginseng medicinal materials. In this research, the spectrum–effect relationship, target cell extraction, and BP neural network classification were used to establish a rapid screening system for potential active substances. The results show that red ginseng extract (RGE) can improve the decrease in cell viability and ATP content and inhibit the increase in ROS production and LDH release in OGD-induced H9c2 cells. A total of 70 ginsenosides were identified in RGE using HPLC-Q-TOF-MS/MS analysis. Chromatographic fingerprints were established for 12 batches of RGE by high-performance liquid chromatography (HPLC). A total of 36 common ingredients were found in 12 batches of RGE. The cell viability, ATP, ROS, and LDH of 12 batches RGE were tested to establish gray relationship analysis (GRA) and partial least squares discrimination analysis (PLS-DA). BP neural network classification and target cell extraction were used to narrow down the scope of Spectral efficiency analysis and screen the potential active components. According to the cell experiments, RGE can improve the cell viability and ATP content and reduce the oxidative damage. Then, seven active ingredients, namely, Ginsenoside Rg1, Rg2, Rg3, Rb1, Rd, Re, and Ro, were screened out, and their cardiovascular activity was confirmed in the OGD model. The seven ginsenosides were the main active substances of red ginseng in treating myocardial injury. This study offers a reference for quality control in red ginseng and preparations containing red ginseng for the management of cardiovascular diseases. It also provides ideas for screening active ingredients of the same type of multi-pharmacologically active traditional Chinese medicines.

## 1. Introduction

Ginseng, specifically the root of *Panax ginseng* Meyer, has been widely utilized as both a tonic food and medicine. The most commonly used varieties of ginseng include Asian ginseng, American ginseng, and notoginseng. In Asia, red ginseng is particularly popular. Red ginseng is produced through a steaming or heating process and has been used for over two thousand years in Asian countries as a medicinal remedy. The active constituents of red ginseng include saponins, polyacetylene alcohols, polysaccharides, fatty acids, and peptides [1].

The main active components of red ginseng are ginsenosides. In recent years, numerous studies have demonstrated the cardiovascular benefits of individual ginsenosides through various mechanisms [2,3,4,5]. These mechanisms include modifying vasomotor function, reducing platelet adhesion, influencing ion channels, altering the release of autonomic neurotransmitters, involvement in glucose metabolism, antioxidant effects, and increasing cardiac output. Over 100 ginsenosides have been identified and isolated from ginseng products, with most of them exhibiting pharmacological activity.

Many traditional Chinese medicine formulae containing ginseng are clinically used in the treatment of cardiovascular diseases, such as Du Shen Tang, Sheng Mai San, Shen Dong Yin, and so on. A study demonstrated that ginsenosides have ion-channel-regulating properties. Specifically, Ginsenoside Rb1 can promote the release of NO to resist cardiomyocyte hypertrophy and, at the same time, reduce the calcineurin signal transduction pathway, thereby attenuating the expression of NFAT3 and GATA4 transcription factors in cardiomyocytes [6]. Ginsenoside Re can reduce ischemia-reperfusion injury in the heart and delay the development of myocardial fibrosis [7,8]. Ginsenoside Rg3, Rh2, and CK have been shown to inhibit different types of Ca^2+^ channels [9,10]. Ginsenoside Rg1 has the ability to decrease left ventricular hypertrophy and protect cardiomyocytes from oxidative injury through its antioxidative effects [11,12,13]. Moreover, Ginsenoside Rb1, Ginsenoside Rc, and Ginsenoside Re have demonstrated a protective role in umbilical vein endothelial cells [14]. Re has also been shown to protect cardiomyocytes from oxidative damage by scavenging hydroxyl radicals and hydrogen peroxide [15]. However, the specific active constituents responsible for the therapeutic effect of ginseng in treating myocardial damage remain unknown.

Finding active ingredients in herbal medicine is a crucial aspect of modern pharmaceutical research. Herbal medicine has played a significant role throughout human history in the battle against diseases. In the past, when scientific and technological resources were limited, herbs were primarily used based on experience and the accumulation of prior knowledge. However, due to human activities, the overexploitation of natural resources has led to variations in the effects of herbal medicines prescribed in ancient texts [16,17]. Such traditional practices can no longer meet the current requirements for ensuring drug quality and efficacy. Fortunately, with the rapid advancement of science and technology, we now possess numerous reliable techniques and methods to conduct in-depth research on herbal medicine. These advancements enable us to explore and understand the active components within these medicinal plants more comprehensively.

Traditionally, the process of identifying potential bioactive constituents in herbal medicine involved isolating and purifying components and then testing their pharmacological activity. However, this method is relatively inefficient for ginseng. Testing the activity of ginsenosides individually is time-consuming and labor-intensive. Furthermore, the nature of herbal medicine suggests that its activity is a result of the collective action of multiple active ingredients. With the advancement of modern analytical techniques [18], more efficient and rapid methods can now be employed for screening active compounds. To address this challenge, the concept of “spectrum–effect relationship” was proposed [19]. By correlating the fingerprint with bioactivity, it becomes possible to link specific bioactive effects to the corresponding constituents in the fingerprint and establish the connection between spectrum characteristics and bioeffect. The “spectrum–effect relationship” approach is a mathematical method in which the prediction of potential active ingredients is based on calculating the correlation coefficient. However, it is important to note that there may be false positives and omissions in the predictions made using this method.

To achieve more accurate predictions, the industry often combines the spectrum–effect relationship method with other approaches. One such method is target cell extraction, which has been utilized to predict the bioactive components in herbal medicine [20,21]. The principle behind this method is that when an extract of herbal medicine is incubated with cells, its potential active components will interact with cell membranes or enter the cells. These components can then be detected in the extract after the cells have been denatured. While the target cell extraction method may still yield some false positive results, combining the information obtained from this method with the spectrum–effect relationship can effectively narrow down the predicted range.

In recent years, artificial intelligence has developed rapidly, and its efficient information-processing capabilities have also been valued by scientific researchers. Machine learning [22] is a subfield of artificial intelligence that studies the ability of computer systems to improve performance by learning from data rather than by improving programming. The goal of machine learning is to allow computer systems to automatically obtain models, rules, knowledge, and other information from large amounts of data and use this information to predict, make decisions, and solve problems. At present, machine learning has been applied in drug research and the quality evaluation of traditional Chinese medicine, having shown good application prospects [23,24].

Increasing the accuracy of predictions can also be achieved by comparing two spectrum–effect relationship analysis methods. The results obtained from these methods can be cross-validated, providing mutual support. Two commonly used spectrum–effect relationship analysis methods are gray relationship analysis (GRA) and partial least squares discrimination analysis (PLS-DA) [25,26,27]. GRA is a systematic analysis method that offers advantages such as not requiring a large number of samples or extensive computations. PLS-DA is a systematic regression analysis method that can build regression models even when the number of samples is smaller than the number of variables [28,29]. Target cell extraction coupled with the spectrum–effect relationship can successfully predict the active ingredients in traditional Chinese medicine. It has previously been applied in our research group to screen active ingredients of *Scutellaria baicalensis* for the treatment of periodontitis [30]. However, due to the complexity and pharmacological diversity of the constituents of TCM, to use multiple pharmacological activity indicators to construct a spectrum–effect relationship is reasonable. A multi-dimensional spectrum–effect relationship will also increase the amount of data and make it difficult to process the data. In essence, selecting active ingredients from the various components of traditional Chinese medicine is also a problem of classifying activity or activity level. Therefore, this study attempts to introduce machine learning to process the data obtained from the above experiments and algorithms in order to obtain more accurate active substances.

In this study, our objective was to evaluate the effectiveness of red ginseng extract (RGE) and identify its active fraction using the ODG H9c2 cell model. We first established the fingerprint of RGE and then utilized spectrum–effect correlation analysis and target cell extraction to obtain spectral efficacy data and target cell binding data. Then, we used BP neural network classification to process the data and finally select the appropriate active ingredients. Subsequently, we conducted experiments to validate the biological activity of the identified active ingredients. The findings of this study serve as a foundation for understanding the therapeutic potential of red ginseng in cardiovascular treatment and offer insights into the screening of active ingredients in other medications.

## 2. Results and Discussion

### 2.1. Anti-Myocardial Ischemia Effect of RGE

**Figure 1:**
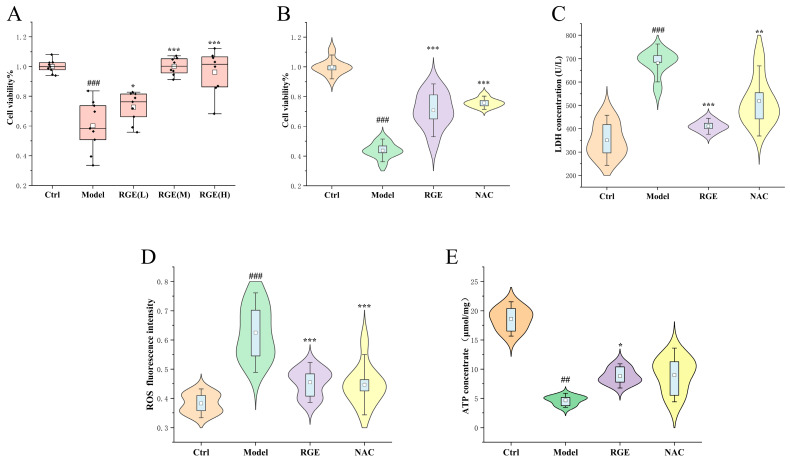
Anti-myocardial ischemia effect of RGE: (**A**) cell viability of different doses of RGE-treated H9c2 cells (*n* = 8) compared with Ctrl and Model group; The box represents the average and the scatter points represent the data distribution. (**B**) cell viability of RGE- and NAC-treated H9c2 cells (*n* = 8) compared with Ctrl and Model group; (**C**) LDH concentration of RGE- and NAC-treated H9c2 cells (*n* = 6) compared with Ctrl and Model group; (**D**) ROS level of RGE- and NAC-treated H9c2 cells (*n* = 6) compared with Ctrl and Model group; (**E**) ATP concentration of RGE- and NAC-treated H9c2 cells (*n* = 3) compared with Ctrl and Model group. Results were presented as mean ± SD., ## *p* < 0.01, ### *p* < 0.001 vs. Ctrl; * *p* < 0.05, ** *p* < 0.01, *** *p* < 0.001 vs. Model. NAC = *N*-acetyl-l-cysteine (positive control).

In this study (Figure 1A,B), the viability of H9c2 cells was assessed using the MTT assay after pre-treatment with three different doses of RGE and NAC, followed by stimulation with OGD injury. The OGD injury model was successfully established by comparing the cell viability of the control group with the OGD-induced group. The results demonstrated that OGD injury significantly inhibited the vitality of H9c2 cells, while treatment with RGE was able to counteract this damage.

Similar trends were observed in the detection of LDH release, ATP content, and ROS production (Figure 1B–D). Compared to the control cells, the OGD-induced cells exhibited increased LDH release, decreased ATP content, and increased ROS production. However, after treatment with 100 μg/mL RGE, the cell viability, LDH release, ATP content, and ROS production approached levels similar to those of the normal group.

These findings indicate that RGE intervention can improve these indicators, suggesting that red ginseng has the ability to combat oxidative damage, improve intracellular energy metabolism levels, enhance cell vitality under pathological conditions, and inhibit the release of myocardial injury markers.

### 2.2. HPLC Fingerprint Analysis and LC-MS Identification

**Figure 2:**
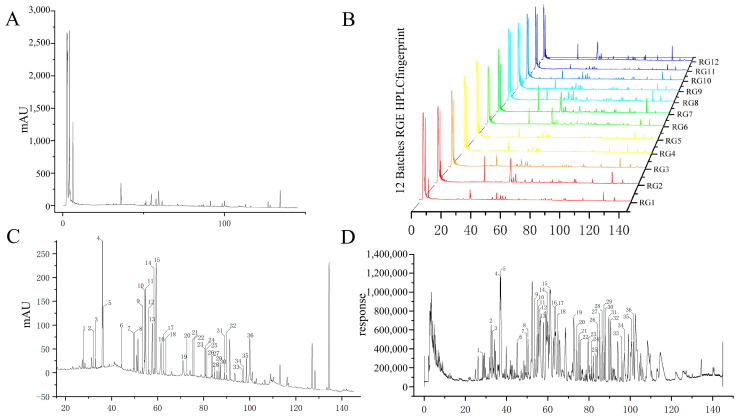
HPLC fingerprint analysis and HPLC-Q-TOF-MS identification. (**A**) HPLC fingerprint of red ginseng extract. (**B**) HPLC fingerprint of 12 batches of red ginseng extract. (**C**) Common peaks of 12 batches of RGE in the HPLC fingerprint. (**D**) Common peaks of 12 batches of RGE in HPLC-Q-TOF-MS.The number represents the serial number of the substance, which can be found in Table S2 and corresponds one to one.

In this study, the HPLC fingerprints of the RGE sample and 12 batches of RGE were generated and compared. The HPLC fingerprints are shown in Figure 2A,B. By analyzing the ultraviolet (UV) spectra and HPLC retention time, 36 common peaks were identified among the 12 batches of RGE samples. These common peaks are marked in Figure 2C to illustrate their presence across multiple batches.

Furthermore, the LC-MS spectrum of the RGE sample was obtained using the Agilent Mass Hunter workstation, as shown in Figure 2D. Through HPLC-Q-TOF-MS analysis, the full-spectrum information was displayed as shown in Table S1. A total of 36 common components of ginsenosides were identified in the RGE sample. Detailed information about these components can be found in Table S2.

This analysis provides valuable insights into the composition and consistency of ginsenosides in different batches of RGE, which is crucial for establishing the spectrum–effect relationship and understanding the potential bioactive components of red ginseng extract.

### 2.3. GRA Spectrum–Effect Relationship Assay

In this study, GRA was conducted to assess the correlation between the spectrum of RGE and its bioactivity. The common compounds identified in the RGE samples were numbered as peak 1, peak 2, peak 3, and so on, based on their retention time. The effects of the 12 batches of RGE on MTT, LDH, ATP, and ROS were determined and presented as mean ± SD in Table S3.

**Figure 3:**
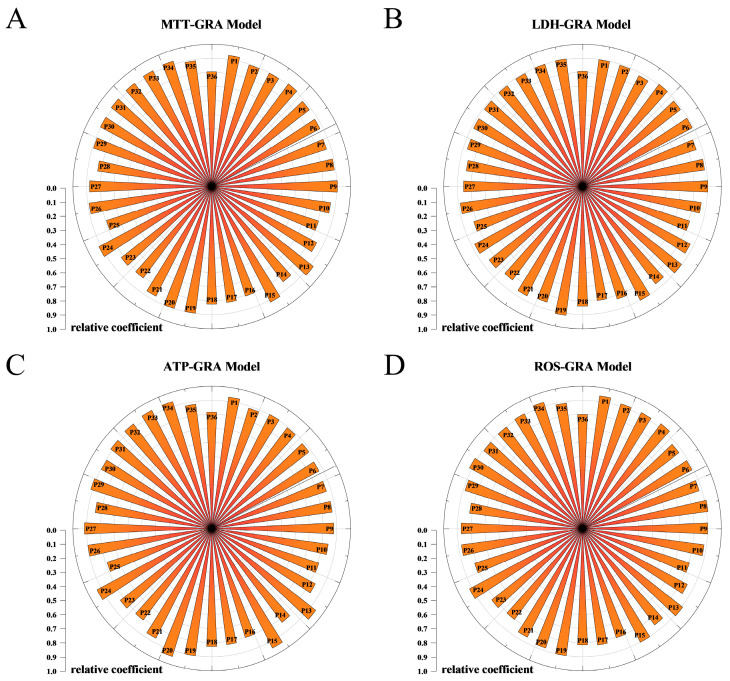
GRA analysis of the multi-anti-myocardial ischemia effect with common peaks of 12 red ginseng batches. (**A**) GRA score of cell viability and common peaks; (**B**) GRA score of LDH level and common peaks; (**C**) GRA score of ATP level and common peaks; (**D**) GRA score of ROS level and common peaks.

Using GRA, the correlation coefficients between the RGE spectrum and bioactivity were calculated. Peaks with correlation coefficients greater than 0.9 were considered to have a high contribution to the efficacy. According to the results, peaks 19, 4, 35, 9, 15, and 34 were found to have a significant contribution to the effect in the LDH-GRA model. In the ATP-GRA and ROS-GRA models, 13 common peaks showed a correlation coefficient, while in the MTT-GRA model, 11 common peaks demonstrated a correlation coefficient (Figure 3).

These findings indicate that specific peaks in the HPLC fingerprint of RGE are closely related to its bioactivity, as indicated by the effects on LDH release, ATP production, ROS levels, and cell viability. These identified peaks can serve as potential markers for evaluating the quality and efficacy of RGE in future studies.

### 2.4. PLS-DA Spectrum–Effect Relationship Analysis

**Figure 4:**
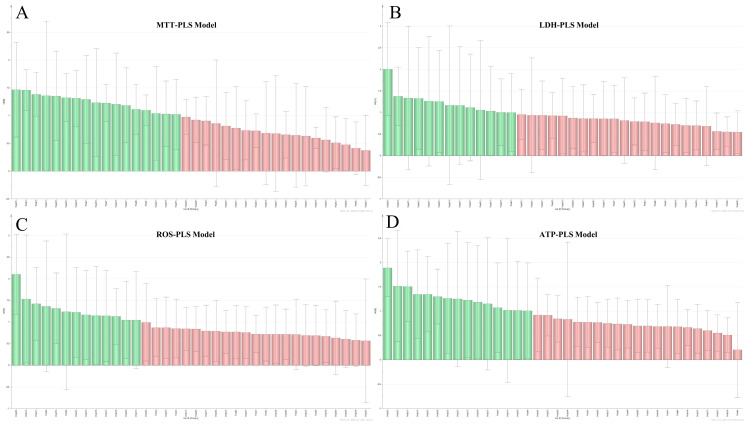
PLS analysis of the multi-anti-myocardial ischemia effect with common peaks of 12 red ginseng batches. (**A**) VIP score of cell viability and common peaks; (**B**) VIP score of LDH level and common peaks; (**C**) VIP score of ATP level and common peaks; (**D**) VIP score of ROS level and common peaks.

In order to further analyze the relationship between the common peaks of the 12 batches of RGE samples and the bioactivity indicators (MTT, LDH, ATP, ROS), PLS-DA was employed (Figure 4). In the PLS-DA model, variables with a variable importance for the projection (VIP) value greater than 1.0 were considered to have a significant contribution to the evaluation of pharmacodynamics.

The results of the PLS-DA analysis revealed that 15 common peaks in the ROS-PLS model had VIP scores exceeding 1.0. Similarly, in the MTT-PLS model, 17 common peaks were identified as having significant contributions. In the LDH-PLS and ATP-PLS models, 13 peaks and 15 peaks, respectively, were considered to be relevant.

These varying results from different PLS models highlight the complex nature of herbal medicine, which typically contains multiple components and targets. Each bioactivity indicator may be influenced by a different set of compounds, leading to differences in the significant peaks identified in each model. Therefore, considering multiple PLS models can provide a more comprehensive understanding of the relationship between the common peaks of RGE and its bioactivity.

### 2.5. Conditions of Cell Extraction

**Figure 5:**
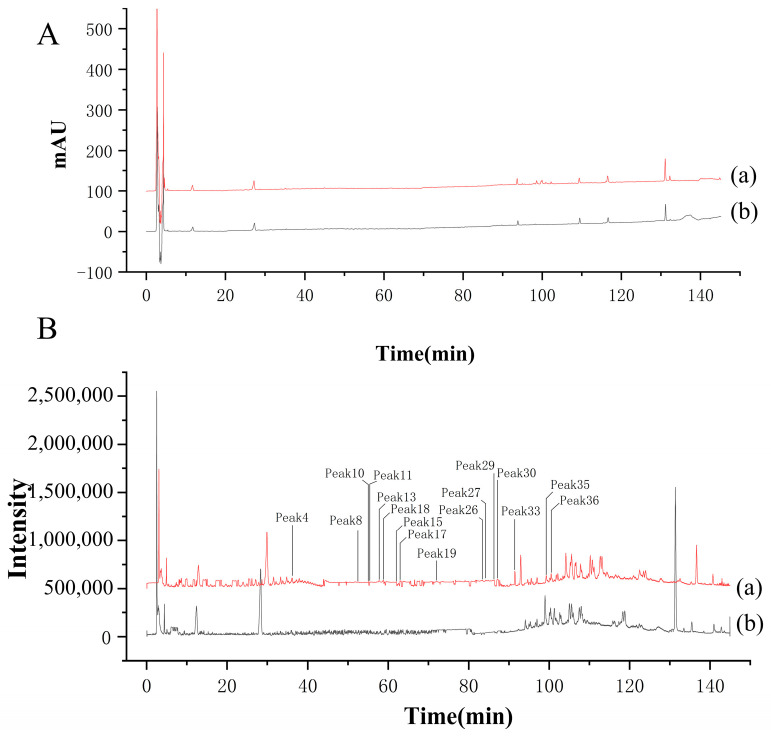
Investigation of cell extraction conditions and cell extraction results. (**A**) The investigation of the number of washings. (a) Fingerprint analysis of cell extraction. (b) Fingerprint analysis of the third PBS washing solution. (**B**) The total ion chromatograms of RGE-H9c2 cell binding molecules by LC-Q-TOF-MS/MS in negative mode. (a) Co-incubation of OGD-induced H9c2 cells and RGE. (b) OGD-induced only.

The number of washings with PBS was investigated to determine the optimal washing protocol for removing unbound components and minimizing interference. It was observed that three times washing with PBS was sufficient to suppress interference and remove unbound constituents (Figure 5A). After the final washing, methanol was used to collect the cells and disrupt them using an ultrasonic cell disruption device. The resulting extract solution was then condensed for HPLC-Q-TOF-MS analysis.

Table S4 shows that 17 constituents of RGE were identified as combined components with the target cells (Figure 5B). These constituents likely play a role in the observed effects on cell viability and other bioactivity indicator.

### 2.6. Target Cell Extraction and Spectrum–Effect Analysis Coupled with BP Neural Network Classification: Verifying the Efficacy of Potential Active Ingredients

**Figure 6:**
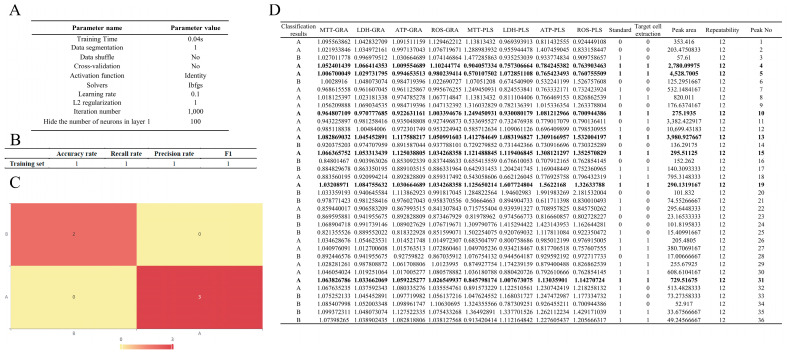
BP neural network classification parameters and results. (**A**) BP neural network classification parameters. (**B**) Training set model evaluation results. (**C**) Confusion matrix heat map, A and B means different classification, the rows represent true classification and the columns represent predict classification. (**D**) BP neural network classification results.

According to the results of target cell extraction and the spectrum–effect analysis, a total of 17 constituents were detected as potentially active compounds of red ginseng. Further analysis using GRA and PLS models predicted more than 20 potential bioactive compounds. Using a single indicator to construct a spectrum–activity relationship can easily obtain information on compounds with a high correlation. However, for traditional Chinese medicine ingredients, multiple components corresponding to multiple activities are the characteristics of traditional Chinese medicine that exert their efficacy. Therefore, in this study, four pharmacodynamic indicators with different mechanism principles were used to construct a spectrum–effect relationship to more comprehensively screen potential active ingredients. However, this also increases the amount of data that needs to be processed. The introduction of a BP neural network classification can better process the obtained data (Figure 6A–C). Considering the content of each compound in the red ginseng extract and its contribution to the spectrum–effect relationship, some compounds with low content were excluded from the selection process. Ultimately, Ginsenoside Rg1, Rg2, Rg3, Rb1, Rd, Re, and Ro were identified as the potential active compounds of red ginseng (Figure 6D).

**Figure 7:**
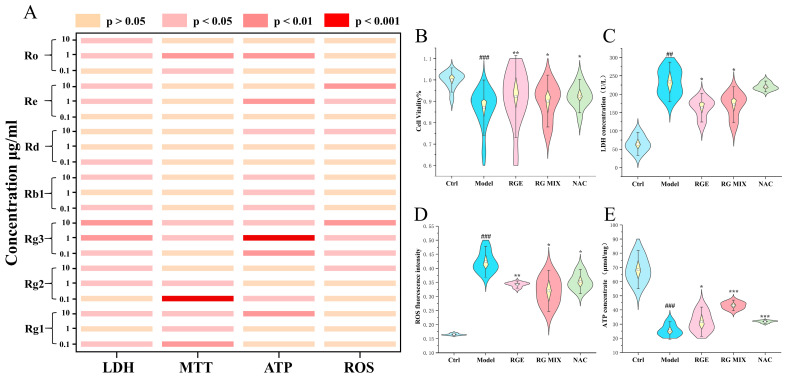
Activity verification of ginsenosides: (**A**) Cell viability, LDH concentration, ROS level, and ATP concentration of single ginsenoside with 3 doges (0.1, 1, 10 μg/mL). (**B**) Cell viability of RGE-, RG MIX (Ginsenoside Rg1, Rg2, Rg3, Rb1, Rd, Re, and Ro)-, and NAC-treated H9c2 cells (*n* = 6) compared with Ctrl and Model group. (**C**) LDH concentration of RGE-, RG MIX (Ginsenoside Rg1, Rg2, Rg3, Rb1, Rd, Re, and Ro)-, and NAC-treated H9c2 cells (*n* = 6) compared with Ctrl and Model group. (**D**) ROS level of RGE-, RG MIX (Ginsenoside Rg1, Rg2, Rg3, Rb1, Rd, Re, and Ro)-, and NAC-treated H9c2 cells (*n* = 6 compared with Ctrl and Model group). (**E**) ATP concentration of RGE-, RG MIX (Ginsenoside Rg1, Rg2, Rg3, Rb1, Rd, Re, and Ro)-, and NAC-treated H9c2 cells (*n* = 3) compared with Ctrl and Model group. Results are presented as mean ± SD. ## *p* < 0.01, ### *p* < 0.001 vs. Ctrl; * *p* < 0.05, ** *p* < 0.01, *** *p* < 0.001 vs. Model. NAC = *N*-acetyl-l-cysteine (positive control).

To validate the activity of these compounds, an activity verification was performed. The heatmap analysis (Figure 7A) showed the significance of the anti-OGD-injury effect, where the depth of color represented the strength of the effect. The RG mix group, composed of the seven selected saponins according to the content of RGE, was able to reduce oxidative damage, increase ATP levels, improve cell viability, and inhibit LDH release, similar to the effects observed with RGE (Figure 7B–D).

### 2.7. Discussion

The results highlight the necessity of combining two spectrum–effect relationships. For example, Ginsenoside Rg1 showed a high score in the GRA model for LDH, MTT, and ROS but had a low relative coefficient in all PLS models. This indicates that Ginsenoside Rg1 can reduce LDH release, improve cell viability, and increase ATP content. The GRA model accurately predicted the strong correlation of Rg1 with LDH, MTT, and ROS, which aligned well with the experimental results. This example illustrates the importance of combining both spectrum–effect relationships. Different calculation principles of the analytical methods can yield distinct results, and a potential active substance may not show consistent response in both methods, potentially leading to its omission if only one spectrum–effect method is used.

The combination of different methods in this study showcased their complementarity and necessity. Target cell extraction yielded 17 chemical components, indicating binding information but not guaranteeing activity. Thus, spectrum–effect analysis was introduced to predict activity. For example, NR2 and Rb3, which combined with H9c2 cells, did not meet the inclusion criteria in all spectrum analyses. Therefore, the combination of target cell extraction, spectrum–effect analysis, and BP neural network classifications can effectively predicted binding components and narrowed down the scope. Different spectrum–effect relationships served to confirm each other and check for gaps. Gray relational analysis alone would have missed Rg1 and Re, while partial least squares discrimination analysis alone would have missed Rb1. In summary, the combination of spectrum–effect analysis, target cell extraction, and BP neural network classifications enabled rapid prediction narrowing, while different spectrum analyses complemented each other.

Overall, the combination of target cell extraction, spectrum–effect analysis using GRA and PLS models, BP neural network classification, and subsequent pharmacological activity verification proved to be an effective approach for identifying and validating the potential active compounds in red ginseng.

## 3. Materials and Methods

### 3.1. Red Ginseng Samples

Red ginseng was purchased from the Tong Ren Tang Chinese Medicine Room in Beijing, China. Prior to HPLC and HPLC-Q-TOF-MS analysis, both the samples and solvents were filtered through a 0.22 μm filter membrane. H9c2 cells were acquired from the Shanghai Chinese Academy of Sciences in Shanghai, China. 3-(4,5-Dimethyl-2-thiazolyl)-2,5-diphenyl-2-*H*-tetrazolium bromide (MTT) and dimethyl sulfoxide (DMSO) were purchased from Titan in Shanghai, China.

### 3.2. Cell Culture

H9c2 cells were cultured in DMEM supplemented with 10% FBS and 1% antibiotics. The cells were maintained in a humidified incubator with 5% CO_2_ and 95% air at a temperature of 37 °C. Prior to use in experiments, the cells were allowed to reach 80–90% confluence in the culture dish.

### 3.3. Oxygen-Glucose Deprivation (OGD) Model and Experimental Groups

H9c2 cells were cultured in dishes until they reached the logarithmic phase of growth. To initiate the experiments, the cells were detached using trypsin and plated in 96/6-well microplates. The cells in the microplates were then subjected to oxygen-glucose deprivation (OGD) for a duration of 14 h [31,32]. OGD injury was induced using non-glucose DMEM and a hypoxic environment consisting of 94% N_2_, 5% CO_2_, and 1% O_2_.

The experiment to confirm the efficacy of the drug involved four groups: the control group (no OGD exposure); the model group (OGD exposure); various concentrations of RGE (25, 100, and 400 μg/mL); and the positive control group (NAC 500 μg/mL). In RGE Groups and positive control group, the RGE and NAC were dissolved in non-glucose DMEM. In the revalidation experiment, Ginsenoside Rg1, Rg2, Rg3, Rb1, Rd, Re, and Ro were individually tested for their anti-OGD injury effects. Additionally, a mixture of these ginsenosides was tested based on their relative content in RGE.

### 3.4. Selection of Detection Indicators and Experimental Methods

Based on the literature review on the cardiovascular protection provided by red ginseng, it has been observed that ginsenosides primarily protect cells by reducing oxidative damage to cardiomyocytes during hypoxic conditions and enhancing the energy supply to these cells. This protection is evident through increased cell viability and reduced release of the myocardial injury marker LDH. To confirm the efficacy of red ginseng extract, four indicators were selected: cell viability, LDH levels, ATP production, and ROS levels. These indicators were utilized to establish the spectrum–effect relationship, which enables a comprehensive screening of potential active substances in red ginseng for cardiovascular protection.

Cell viability was assessed using the MTT assay. H9c2 cells were seeded at a density of 6000 cells/well in a 96-well microplate. After the induction of OGD injury and treatment in each group, the cell culture medium was collected for LDH activity assay using a kit, following the manufacturer’s instructions. Next, MTT solution (0.5 mg/mL) was added to each well, and they were incubated for an additional 4 h. The MTT solvent was then discarded, and dimethyl sulfoxide (DMSO) was added at a volume of 150 μL/well. The absorbance was measured at a wavelength of 570 nm (with a reference wavelength of 650 nm) using a spectrophotometer.

For the measurement of ATP production, H9c2 cells were plated in 6-well microplates. After the induction of injury and treatment, cells were collected from the 6-well plate and washed twice. The cells were then lysed using an ATP extracting solution, and the resulting supernatant was subjected to ATP detection using luminometric methods.

In the measurement of ROS levels, cells in the 96-well plates were utilized. The supernatant was discarded, and 2′,7′-dichlorodihydrofluorescein diacetate (DCFH-DA) was added to the cells. The plate was then incubated at 37 °C for 20 min. After the incubation period, the cells were washed with non-FBS DMEM and PBS, and the fluorescence was determined using a fluorescence microplate reader (BioTek Instruments, Winooski, VT, USA).

### 3.5. RGE Preparation and HPLC-MS Analysis

Twelve batches of red ginseng were purchased from the TRT Chinese Medicine Room and were authenticated by Professor Boyang Yu of China Pharmaceutical University. These twelve batches of red ginseng have different production batch numbers. The composition differences and content differences between different batches are the foundation of spectrum–effect relationships.

In the experiment, all red ginseng samples were milled into 60-mesh powders. One gram of the powdered sample was accurately weighed and subjected to extraction in a water bath at 75 °C. The extraction process was carried out by refluxing and extracting three times using ethanol–water (75:25) as the solvent. The dissolvent containing the extracted components was collected, filtered, and concentrated to a final volume of 5 mL. The resulting RGE samples were then filtered through a 0.22 μm membrane for further analysis using HPLC and HPLC-MS.

The chromatographic separation of the RGE samples was performed using an Agilent 1260 Infinity HPLC system (Santa Clara, CA, USA) equipped with a Kromasil 100-5-C18 column (4.6 mm × 250 mm, 5 μm) (Nouryon, Stenungsund, Sweden). The mobile phase consisted of two components: 0.01% methane acid (A) and 0.01% methane acid acetonitrile (B). A gradient elution method was employed with a total duration of 145 min. The optimized HPLC program included the following steps: 0–15 min, 18% solvent A and 82% solvent B; 15–25 min, 18–22% solvent A and 82–78% solvent B; 25–45 min, 22–32% solvent A and 78–68% solvent B; 45–65 min, 32–34% solvent A and 68–66% solvent B; 65–75 min, 34–42% solvent A and 66–58% solvent B; 75–95 min, 42–60% solvent A and 58–40% solvent B; 95–110 min, 60–70% solvent A and 40–30% solvent B; 110–135 min, 70–95% solvent A and 30–5% solvent B; 135–145 min, 95% solvent A and 5% solvent B. The separation of RGE was performed at a constant flow rate of 0.8 mL/min, and 20 μL of the RGE solution was injected for analysis. The column oven temperature was set to 35 °C.

For mass spectrometry analysis, an Agilent 6530 Q-TOF-MS/MS mass spectrometer with an electron spray ionization (ESI) source was used to collect the mass spectrometry data. The acquired data were analyzed using the Agilent Mass Hunter workstation qualitative analysis software version B.06.00.

### 3.6. Target Cell Extraction

In the experiment, H9c2 cells were cultured in 20 cm culture dishes and divided into three groups: the control, the model, and the RGE cell extraction group. In the RGE group, the cells were treated with RGE at a concentration of 10 mg/mL. Both the model and RGE groups were exposed to an OGD environment for 4 h to induce the desired conditions.

After the OGD exposure, the culture medium was discarded, and the cells were washed with PBS (phosphate-buffered saline) to remove any uncombined constituents. The PBS used for washing was collected for further analysis. Methanol was then added to the dish to collect the cells that had combined with the potential activity components present in the RGE. An ultrasonic cell disruption device was used to disrupt the cells and release the components that were bound to the H9c2 cells. The disrupted cells were subjected to cryogenic centrifugation at 12,000× *g* for 20 min. The resulting supernatant, containing the released components, was concentrated to a volume of 200 μL for subsequent HPLC-MS analysis.

### 3.7. Spectrum–Effect Relationship between HPLC Fingerprints and Anti-OGD Injury Effect of Red Ginseng

In this study, the HPLC chromatography method described in Section 3.5 was employed to analyze multiple batches of red ginseng samples. All 15 batches of red ginseng powder were dissolved in DMEM medium and diluted to a concentration of 100 μg/mL. The anti-myocardial ischemia effect of these 15 batches of RGE was then evaluated in vitro using H9c2 cells.

Several parameters, including cell viability, LDH release, ATP production, and ROS level, were used to establish the spectrum–effect relationship between the chromatographic profile of red ginseng samples and their anti-myocardial ischemia activity.

To calculate the correlation coefficient between the chromatographic profile and the anti-myocardial ischemia effect of RGE, GRA and PLS were employed. These statistical methods were used to determine the degree of correlation between the chromatographic characteristics of the red ginseng samples and their efficacy in protecting against myocardial ischemia.

### 3.8. The Data Processing BP Neural Network Classification

The spectral efficiency data obtained from the four activity indicators and the target cell binding data are included in the BP neural network classification data set, and the classification results are outputted.

## 4. Conclusions

In this study, the protective effects of RGE on H9c2 cells under hypoxic conditions were investigated. The aim was to reduce oxidative damage to cardiomyocytes and enhance their energy supply, leading to increased cell viability and decreased release of the myocardial injury marker LDH. Multiple indicators including MTT, LDH, ROS, and ATP were chosen to establish a spectrum–effect relationship. Additionally, affinity chromatography was employed to obtain information on the combined constituents of RGE, resulting in the selection of 17 ginsenosides through target cell extraction.

Spectrum–effect analysis provided correlation coefficients for each peak, allowing for the identification of quality control constituents of red ginseng. Compounds with high correlation coefficients and combined information were chosen as potential active ingredients. The combined approach involved the establishment of four GRA models and four PLS models to predict the potential effect constituents of red ginseng. Using BP neural network classification to process the above data, ultimately, seven constituents (Ginsenoside Rg1, Rg2, Rg3, Rb1, Rd, Re, and Ro) were identified as the potential active ingredients of RGE, which demonstrated bioactivity against OGD-induced damage in H9c2 cells. These components hold reference value for the quality control of red ginseng and its prescription in cardiovascular diseases.

This study provides a viable research model for the rapid discovery of active components in other herbal medicines.

## Data Availability

The data that support the findings of this study are openly available in 4tu.researchdata.

## Supplementary Materials

The following supporting information can be downloaded at https://www.mdpi.com/article/10.3390/molecules29092028/s1, Table S1. LC–MS/MS data in the negative ion mode for all compounds identified in RGE. Table S2. LC–MS/MS data in the negative ion mode for common peaks identified in 12 batches RGE. Table S3. Anti-Myocardial ischemia effect of 12 batches RGE (mean ± SD). Table S4. LC–MS/MS data in the negative ion mode for identification of target cell extraction combined with cells. Table S5. The result of GRA model and PLS model.

## Abbreviations

OGD	Oxygen-glucose deprivation
HPLC	High-performance liquid chromatography
GRA	Gray relationship analysis
MTT	3-(4,5-Dimethyl-2-thiazolyl)-2,5-diphenyl-2-*H*-tetrazolium bromide
DMEM	Dulbecco’s modified Eagle’s medium
FBS	Fetal bovine serum
NAC	*N*-Acetyl-l-cysteine
ROS	Reactive oxygen species
DCFH-DA	2′,7′-Dichlorodihydrofluorescein diacetate
ESI	Electron spray ionization
UV	Ultraviolet
RG	Red ginseng
RGE	Red ginseng extract
PLS-DA	Partial least squares discrimination analysis
DMSO	Dimethyl sulfoxide
NO	Nitric oxide
VIP	Variable importance for the projection
ATP	Adenosine triphosphate
LDH	Lactic dehydrogenase
HPLC-MS	High-performance liquid chromatography tandem mass spectrometry
PBS	Phosphate-buffered saline
