# Supplementary material for: Target Cell Extraction and Spectrum–Effect Relationship Coupled with BP Neural Network Classification for Screening Potential Bioactive Components in Ginseng Extract with a Protective Effect against Myocardial Damage
Source: Molecules. 2024 Apr 28;29(9):2028. doi: 10.3390/molecules29092028 (PMC11085743; doi:10.3390/molecules29092028)
Supplement: Supplementary file 1 [file molecules-29-02028-s001.zip › molecules-2943800-supplementary.pdf]

Table S1. LC–MS/MS data in the negative ion mode for all componds identified in RGE

No	IDENTIFICATION	RT	FORMULE	M/Z	M/Z CAL	PPM	IONS
1	Rg12/Re5/Ginsenjilinol/f-G-E/f-G-F	24.847	$C_{42}H_{72}O_{15}$	861.48.27	1252	1	861.4854[M-H+HCOOH]$^-$;815.4796[M-H]$^-$;653.4202[M-Glc-H]$^-$;491.3760[M-2Glc-H]$^-$
2	Rg12/Re5/Ginsenjilinol/f-G-E/f-G-F	26.185	$C_{42}H_{72}O_{15}$	861.4848	861.4853	0.61	861.4827[M-H+HCOOH]$^-$;815.4760[M-H]$^-$;653.4244[M-Glc-H]$^-$;491.3728[M-2Glc-H]$^-$
3	Noto ginsenoside R3	29.195	$C_{48}H_{82}O_{19}$	1007.5424	1007.5358	2.77	1007.5375[M-H+HCOOH]$^-$;961.5355[M-H]$^-$; 799.4811[M-Glc-H]$^-$;637.4298[M-2Glc-H]$^-$; 475.3744[M-3Glc-H]$^-$
4	Re4/Q-F6	31.804	$C_{47}H_{80}O_{18}$	977.5325	977.5327	0.17	977.5325[M-H+HCOOH]$^-$;931.5250[M-H]$^-$;799.4843[M-Arap-H]$^-$;637.4276[M-Arap-Glc-H]$^-$; 475.3744[M- Arap -2Glc-H]$^-$
5	G-Ki/G-Km/G-ST2	32.339	$C_{36}H_{62}O_{10}$	699.4337	699.4325	1.71	699.4322[M-H+HCOOH]$^-$; 689.4058[M-H+Cl]$^-$; 653.4281[M-H]$^-$;491.3749[M-Glc-H]$^-$
6	Noto ginsenoside R1	33.208	$C_{47}H_{80}O_{18}$	977.5739	977.5327	3.47	977.5332[M-H+HCOOH]$^-$;931.5259[M-H]$^-$;799.4872[M-Arap-H]$^-$;637.4310 [M- Arap -Glc-H]$^-$
7	Re1/Re2/Re3/NG-N/VG-R4	33.476	$C_{48}H_{82}O_{19}$	1007.5456	1007.5432	-2.35	1007.5420[M-H+HCOOH]$^-$;961.5366[M-H]$^-$;799.4791[M-Arap-H]$^-$;637.4272 [M- Arap -Glc-H]$^-$
8	Re4/Q-F6	34.011	$C_{47}H_{80}O_{18}$	977.5343	977.5327	-1.67	977.5313[M-H+HCOOH]$^-$;931.5248[M-H]$^-$;799.4786[M-Arap-H]$^-$;637.4297[M- Arap -Glc-H]$^-$;475.3810[M- Arap -2Glc-H]$^-$

No	IDENTIFICATION	RT	FORMULE	M/Z	M/Z CAL	PPM	IONS
9	Re1/Re2/Re3/NG-N/VG-R4	34.546	$C_{48}H_{82}O_{19}$	1007.5429	1007.5432	0.33	1007.5429[M-H+HCOOH]$^-$;961.5356[M-H]$^-$; 799.4787[M-Arap-H]$^-$;637.4008 [M- Arap -Glc-H]$^-$; 475.3786[M- Arap -2Glc-H]$^-$
10	Rg12/Re5/Ginsenjilinol/f-G-E/f-G-F	34.747	$C_{42}H_{72}O_{15}$	861.4856	861.4853	-0.32	861.4856[M-H+HCOOH]$^-$;815.4786[M-H]$^-$;653.4244[M-Glc-H]$^-$;491.3659 [M-2Glc-H]$^-$
11	Re1/Re2/Re3/NG-N/VG-R4	35.683	$C_{48}H_{82}O_{19}$	1007.5434	1007.5432	-0.17	1007.5459[M-H+HCOOH]$^-$;961.5384[M-H]$^-$;799.4892[M-Glc-H]$^-$;637.4263[M-2Glc-H]$^-$;475.3660[M-3Glc-H]$^-$
12	Ginsenoside Rg1	36.887	$C_{42}H_{72}O_{14}$	845.5235	845.5243	0.14	845.5235[M-H+HCOOH]$^-$;637.4520[M-H-Glc]$^-$; 475.3940[M-H-2Glc]$^-$
13	Ginsenoside Re	36.564	$C_{48}H_{82}O_{18}$	991.5876	991.5893	1.99	991.5876[M-H+HCOOH]$^-$;945.5802[M-H]$^-$; 799.5148[M-H-Rha]$^-$; 637.4528[M-H--Rha Glc]$^-$;475.3901[M-H-Rha-2Glc]$^-$
14	Malony(丙二酰)-Re	41.971	$C_{51}H_{84}O_{21}$	1031.5386	1031.5432	4.49	1031.5386[M-H]$^-$;885.4799[M-H-Rha]$^-$; 739.1880[M-H-2Rha]$^-$;739.1880[M-H-2Rha-Arap]$^-$
15	Ac-Rd	42.038	$C_{50}H_{84}O_{19}$	987.5502	987.5475	-2.7	987.5475[M-H]$^-$;841.4872[M-H-Rha]$^-$;679.3690[M-Glc-Rha-H]$^-$;491.3659 [M-2Glc-H]$^-$
16	NG-R2/G-F3/G-F5	44.112	$C_{41}H_{70}O_{13}$	815.4786	815.4798	1.89	815.4788[M-H+HCOOH]$^-$;769.4716[M-H]$^-$; 637.4225[M-H-Arap]$^-$;
17	NG-R2/G-F3/G-F5	45.048	$C_{41}H_{70}O_{13}$	815.4769	815.4798	3.61	815.4791[M-H+HCOOH]$^-$;769.4724[M-H]$^-$; 637.4288[M-H-Arap]$^-$; 475.3750[M-H-Arap-Glc]$^-$;

No	IDENTIFICATION	RT	FORMULE	M/Z	M/Z CAL	PPM	IONS
18	Notoginsenoside Rt	45.115	$C_{44}H_{74}O_{15}$	887.4717	887.4567	0.62	887.4717[M-H+HCOOH]$^-$;841.4797[M-H]$^-$; 637.4119[M-H-C2H2O-Glc]$^-$;475.3662[M-H-C2H2O-2Glc]$^-$
19	Re1/Re2/Re3/NG-N/VG-R4	47.055	$C_{48}H_{82}O_{19}$	1007.5373	1007.5432	5.88	1007.5373[M-H+HCOOH]$^-$;961.5232[M-H]$^-$;799.4696[M-Glc-H]$^-$;637.4309[M-2Glc-H]$^-$;475.2728[M-3Glc-H]$^-$
20	Rg12/Re5/Ginsenjilinol/f-G-E/f-G-F	47.590	$C_{42}H_{72}O_{15}$	861.4860	861.4853	-0.78	861.4864[M-H+HCOOH]$^-$;815.4808[M-H]$^-$;653.4232[M-Glc-H]$^-$;491.3730[M-2Glc-H]$^-$
21	Ginsenoside Ra3/R4	49.673	$C_{59}H_{100}O_{27}$	1239.6907	1239.6902	0.5	1239.6907[M-H]$^-$;1077.6448[M-H-Xly]$^-$;945.5900[M-H-Xly-Glc]$^-$;783.5144[M-H-Xly-2Glc]$^-$;621.4648[M-H-Xly-3Glc]$^-$;459.3907[M-H-Xly-4Glc]$^-$
22	Re1/Re2/Re3/NG-N/VG-R4	50.868	$C_{48}H_{82}O_{19}$	1007.5428	1007.5432	0.73	1007.5412[M-H+HCOOH]$^-$;961.5358[M-H]$^-$;799.4867[M-Glc-H]$^-$;637.4200[M-2Glc-H]$^-$;475.3823[M-3Glc-H]$^-$
23	Ginsenoside Rf	52.406	$C_{42}H_{72}O_{14}$	845.5204	845.4904	2.36	845.5204[M-H+HCOOH]$^-$;799.5129[M-H]$^-$;637.4518[M-H-Glc]$^-$;475.3920[M-H-2Glc]$^-$;391.2939[M-H-2Glc-C6H12]$^-$; 221.0694 1,3A2β； 101.0239 2,5A1β
24	Ginsenoside Rf isomer	53.677	$C_{42}H_{72}O_{14}$	845.4917	845.4904	-1.52	845.4957[M-H+HCOOH]$^-$;799.4869[M-H]$^-$; 637.4304[M-H-Glc]$^-$;475.3801[M-H-2Glc]$^-$
25	Ginsenoside Ra2/Ra1	54.814	$C_{58}H_{98}O_{26}$	1209.6777	1209.6732	2.02	1209.6282[M-H]$^-$;915.5354[M-Glc-Xylp-H]$^-$;783.5187[M-Glc-Xylp-Araf-H]$^-$; 621.4573[M-2Glc-Xylp-Araf-H]$^-$; 459.3967[M-3Glc-Xylp-Araf-H]$^-$

No	IDENTIFICATION	RT	FORMULE	M/Z	M/Z CAL	PPM	IONS
26	NG-R2/G-F3/G-F5	55.483	$C_{41}H_{70}O_{13}$	815.4798	815.4798	0.06	815.4798[M-H+HCOOH]$^-$;769.4727[M-H]$^-$; 637.4295[M-H-Arap]$^-$; 475.3791[M-H-Arap-Glc]$^-$;
27	Ginsenoside R4/Ra3	55.818	$C_{59}H_{100}O_{27}$	1239.6385	1239.6379	0.47	1239.6358[M-H]$^-$;1077.5830[M-H-Xly]$^-$;945.5326[M-H-Xly-Glc]$^-$;783.4673[M-H-Xly-2Glc]$^-$;621.4356[M-H-Xly-3Glc]$^-$;459.3962[M-H-Xly-4Glc]$^-$
28	Ginsenoside Rb1	56.152	$C_{54}H_{92}O_{23}$	1107.6414	1107.6426	-3.19	1153.6413[M-H+HCOOH]$^-$;1107.6424[M-H]$^-$; 945.5788[M-H-Glc]$^-$; 783.5190[M-H-2Glc]$^-$; 621.4607[M-H-3Glc]$^-$;459.4013[M-H-4Glc]$^-$
29	m- Rb1	57.825	$C_{57}H_{94}O_{26}$	1193.5951	1193.5961	0.8	1193.5951[M-H]-; 1149.6028[M-H-CO_2]$^-$ 1107.5883[M-H-Ma]$^-$; 945.5371[M-H-Glc-Ma]$^-$; 783.4886[M-H-2Glc-Ma]$^-$; 621.4607[M-H-3Glc]-;459.4013[M-H-4Glc]$^-$
30	Ginsenoside Rg2	59.096	$C_{42}H_{72}O_{13}$	829.4994	829.4955	-4.7	829.4994[M-H+HCOOH]$^-$;783.4933[M-H]$^-$;475.8112[M-H-Rha-Glc]$^-$; 391.9717[M-H-Rha-Glc-C6H12]$^-$
31	Ginsenoside Rb3	59.497	$C_{53}H_{90}O_{22}$	1123.5971	1123.5906	-5.8	1077.5891[M-H]$^-$;945.6899[M-Arap-H]$^-$;783.4933[M-Arap-Glc-H]$^-$; 621.4260[M-Arap-2Glc-H]$^-$;459.3817 [M-Arap-3Glc-H]$^-$
32	Ginsenoside Rc	63.644	$C_{53}H_{90}O_{22}$	1077.6291	1077.6282	5.1	1077.6291[M-H]$^-$;945.5783[M-Araf-H]$^-$;783.5164[M-H-Araf-Glc]$^-$;621.4609[M-H-Araf-2Glc]$^-$;459.3980[M-H-Araf-3Glc]$^-$
33	Rh1	60.032	$C_{36}H_{62}O_9$	683.4383	683.4376	-1.04	683.4377 [M-H+HCOOH]$^-$;637.4321 [M-H]$^-$; 475.3721[M-H-Glc]$^-$
34	Ginsenoside F2	60.567	$C_{42}H_{72}O_{13}$	829.4959	829.4955	-0.49	829.4959 [M-H+HCOOH]$^-$;783.4890 [M-H]$^-$; 637.4297[M-H-Rha]$^-$

No	IDENTIFICATION	RT	FORMULE	M/Z	M/Z CAL	PPM	IONS
35	Ginsenoside Ro	61.169	$C_{48}H_{76}O_{19}$	955.5309	955.5295	5.86	955.5309[M-H]-;793.4684 [M-H-Glc]-;775.4506[M-H-H2O-Glc]-;731.4631[M-H-CO2-H2O-Glc]-; 613.3954[M-H-H2O-2Glc]-;569.4022[M-H-CO2-H2O-2Glc]-;455.3675[M-H-2Glc -GlcA]-
36	m-Rc	61.571	$C_{56}H_{92}O_{25}$	1163.5850	1163.5855	0.42	1163.5850[M-H]-; 1077.6291[M-H-Ma]$^-$ 1001.2398[M-Araf-H]-;783.4866[M-H-Araf-Glc-Ma]-;621.4341[M-H-Araf-2Glc-Ma]-;
37	Ginsenoside F1	62.775	$C_{36}H_{62}O_9$	683.4351	683.4376	3.63	683.4351 [M-H+HCOOH]$^-$;637.2598 [M-H]$^-$; 475.3772[M-H-Glc]$^-$
38	Ginsenoside Rb2	64.915	$C_{53}H_{90}O_{22}$	1123.5948	1123.5906	-3.75	1077.5916[M-H]$^-$;945.5488[M-Arap-H]$^-$;783.4918[M-Arap-Glc-H]$^-$;621.4609[M-Arap-2Glc-H]$^-$; 459.4037[M-Arap-3Glc-H]$^-$
39	Ac-Rc	65.852	$C_{55}H_{92}O_{23}$	1119.5976	1119.5957	-1.73	1119.5927[M-H]$^-$;987.5522[M-Arap-H]$^-$;825.4959[M-Arap-Glc-H]$^-$;663.4123 [M-Arap-2Glc-H]$^-$
40	m-Rb2	65.919	$C_{56}H_{92}O_{25}$	1163.5835	1163.5855	1.71	1163.5835[M-H]$^-$; 1077.5824[M-H-Ma]$^-$;783.4824[M-H-Araf-Glc-Ma]$^-$;621.4389[M-H-Araf-2Glc-Ma]$^-$;
41	m-Rb3	67.123	$C_{56}H_{92}O_{25}$	1163.5864	1163.5855	-0.78	1163.5851[M-H]$^-$; 1077.5838[M-H-Ma]$^-$;783.4909[M-H-Araf-Glc-Ma]$^-$;621.4355[M-H-Araf-2Glc-Ma]$^-$;
42	Ac(乙酰基)-Rb1	68.527	$C_{56}H_{94}O_{24}$	1149.6038	1149.3062	2.11	1149.6067[M-H]$^-$;987.5680[M-Glc-H]$^-$;825.4937[M-2Glc-H]$^-$; 663.4506[M-3Glc-H]$^-$;

No	IDENTIFICATION	RT	FORMULE	M/Z	M/Z CAL	PPM	IONS
43	Rh19	69.932	$C_{36}H_{62}O_9$	683.4369	683.4376	1	683.4367[M-H]$^-$;537.3386[M-Arap-H]$^-$;391.3530[M-Arap-Rha-H]$^-$
44	m-Rc-isomer	71.538	$C_{56}H_{92}O_{25}$	1163.5808	1163.5855	4.03	1163.5808[M-H]-; 1077.5808[M-H-Ma]$^-$;783.4979[M-H-Araf-Glc-Ma]-;621.4227[M-H-Araf-2Glc-Ma]-;
45	Ra5/isomer	72.073	$C_{60}H_{100}O_{27}$	1251.6446	1251.6379	-5.33	1251.6446[M-H]$^-$;1119.5950[M-H-Arap]$^-$; 957.9219[M-H-2Arap]$^-$;825.4944[M-H-3 Arap]$^-$;
46	Ginsenoside Rd	72.541	$C_{48}H_{82}O_{18}$	991.5879	991.5483	-0.59	991.5879[M-H+HCOOH]$^-$;945.5791[M-H]$^-$;783.5167[M-H-Glc]$^-$; 621.4575[M-H-2Glc]$^-$; 459.3973[M-H-3Glc]$^-$; 101.0238 2,5A1α/2,5A1β
47	Maonyl-Ginsenoside Rd	74.08	$C_{51}H_{84}O_{21}$	1031.5451	1031.5432	1.81	1031.5374[M-H]$^-$;945.5426[M-H-Ma]$^-$;783.4961[M-H-Glc-Ma]$^-$;765.4784[M-H-H2O-Glc-Ma]$^-$; 621.4375[M-H-2Glc-Ma]$^-$;459.3864[M-H-3Glc-Ma]$^-$
48	Malony(丙二酰)-Re	74.949	$C_{51}H_{84}O_{21}$	1031.5404	1031.5432	2.74	1031.5362[M-H]$^-$;945.5352[M-H-Ma]$^-$;783.4920[M-H-Glc-Ma]$^-$;765.4708[M-H-H2O-Glc-Ma]$^-$; 621.4300[M-H-2Glc-Ma]$^-$;459.3924[M-H-3Glc-Ma]$^-$
49	Ginsenoside Rs2/ Rs1	75.15	$C_{55}H_{92}O_{23}$	1165.6514	1165.6011	1.84	1165.5949[M-H+HCOOH]$^-$;1119.5988[M-H]$^-$;1077.5854[M-C2H2O-H]$^-$ 945.5429[M-C2H2O-Araf-H]$^-$;915.5755[M-C2H2O-Glc-H]$^-$; 783.5176[M-C2H2O-Glc-Araf-H]$^-$;621.4214[M-C2H2O-2Glc-Araf-H]$^-$; 459.3903[M-C2H2O-3Glc-Araf-H]$^-$

No	IDENTIFICATION	RT	FORMULE	M/Z	M/Z CAL	PPM	IONS
50	Ginsenoside Rs2/ Rs1	75.752	$C_{55}H_{92}O_{23}$	1165.6022	1165.6011	4.32	1165.6514[M-H+HCOOH]$^-$;1119.6416[M-H]$^-$; 1077.6260[M-C2H2O-H]$^-$;945.5444[M-C2H2O-Arap-H]$^-$; 915.5436[M-C2H2O-Glc-H]$^-$; 783.4980[M-C2H2O-Glc-Arap-H]$^-$;621.4163[M-C2H2O-2Glc-Arap-H]$^-$;459.3801[M-C2H2O-3Glc-Arap-H]$^-$
51	Ac-Rb2	76.889	$C_{55}H_{92}O_{23}$	1165.5963	1165.6011	-0.22	1165.6014[M-H+HCOOH]$^-$;1119.5965[M-H]$^-$; 1077.5869[M-C2H2O-H]$^-$;945.5513[M-C2H2O-Arap-H]$^-$; 915.5233[M-C2H2O-Glc-H]$^-$;
52	Ac-Rb3	78.695	$C_{55}H_{92}O_{23}$	1165.5964	1165.6011	4.06	1165.5997[M-H+HCOOH]$^-$;1119.5933[M-H]$^-$; 1077.5825[M-C2H2O-H]$^-$;945.5332[M-C2H2O-Arap-H]$^-$; 915.5251[M-C2H2O-Glc-H]$^-$;
53	Ac-Rd isomer	79.565	$C_{50}H_{84}O_{19}$	987.5466	987.5534	6.88	1033.5532[M-H+HCOOH]$^-$;987.5466[M-H]$^-$; 825.4892[M-H-Glc]$^-$;663.4407[M-H-2Glc]$^-$
54	NG-Fe	79.693	$C_{47}H_{80}O_{17}$	961.5361	961.5378	1.72	961.5324[M-H+HCOOH]$^-$;915.5144[M-H]$^-$; 783.4822[M-H-Arap]$^-$;
55	Ginsenoside Rg9/Rg10	79.966	$C_{42}H_{70}O_{13}$	827.4785	827.4798	0.3	827.4767[M-H+HCOOH]$^-$;781.4759[M-H]$^-$; 619.4167[M-H-Glc]$^-$;457.3706[M-H-2Glc]$^-$
56	VG-R16	80.100	$C_{47}H_{80}O_{17}$	961.5324	961.5378	5.56	961.5360[M-H+HCOOH]$^-$;915.5322[M-H]$^-$; 783.4720[M-H-Arap]$^-$;
57	Ginsenoside Rg9/Rg10	81.304	$C_{42}H_{70}O_{13}$	827.4785	827.4798	0.3	827.4767[M-H+HCOOH]$^-$;781.4718[M-H]-; 619.4133[M-H-Glc]$^-$;457.3612[M-H-2Glc]$^-$
58	Ac-Re isomer	82.040	$C_{50}H_{84}O_{19}$	987.5467	987.5534	6.78	1033.5537[M-H+HCOOH]$^-$;987.5467[M-H]$^-$; 825.4893[M-H-Glc]$^-$;663.4323[M-H-2Glc]$^-$

No	IDENTIFICATION	RT	FORMULE	M/Z	M/Z CAL	PPM	IONS
59	Notoginsenoside T5	82.976	$C_{41}H_{68}O_{12}$	797.4671	797.4693	2.73	797.4642[M-H+HCOOH]-;751.4656[M-H]-; 619.4252[M-H-Xylp]-;457.3645[M-H-Xylp-Glc]-
60	Ginsenoside Rg6/Rg4	84.047	$C_{42}H_{70}O_{12}$	811.4838	811.4849	1.39	811.4791[M-H+HCOOH]-;765.4675[M-H]-; 619.4211[M-H-Rha]-;457.3609[M-H-Glc-Rha]-
61	Ginsenoside Rg6/Rg4	85.251	$C_{42}H_{70}O_{12}$	811.4848	811.4849	1.39	811.4791[M-H+HCOOH]-;765.4859[M-H]-; 619.4211[M-H-Rha]-;457.3609[M-H-Glc-Rha]-
62	Chikusetsusaponin IVa	87.324	$C_{42}H_{66}O_{14}$	793.4369	793.4380	1.36	793.4329[M-H]-;613.3735[M-Glc-H]-;587.3984[M-Glc-CO2-H]-;455.3528[M-Glc-GlcA-H]-
63	Ginsenoside Rh4/Rk3	86.388	$C_{36}H_{60}O_8$	665.4273	665.4270	1.23	665.4235[M-H+HCOOH]-;619.3950[M-H]-; 457.3587[M-H-Glc]-
64	Ginsenoside Rh4/Rk4	87.859	$C_{36}H_{60}O_8$	665.4272	665.4270	-0.27	665.4235[M-H+HCOOH]$^-$;619.3950[M-H]$^-$; 457.3588[M-H-Glc]$^-$
65	20(S)-ginsenoside Rg3	89.665	$C_{42}H_{72}O_{13}$	829.4973	829.4955	-2.17	829.4920[M-H+HCOOH]$^-$;783.4895[M-H]$^-$; 621.4357[M-H-Glc]$^-$; 549.3828[M-H-2Glc]$^-$
66	20(R)-ginsenoside Rg3	90.468	$C_{42}H_{72}O_{13}$	829.4999	829.4955	-5.3	829.4920[M-H+HCOOH]$^-$;783.4897[M-H]$^-$;621.4385[M-H-Glc]$^-$;459.3853[M-H-2Glc]$^-$
67	20(S)-ginsenoside Rs3	96.02	$C_{44}H_{74}O_{14}$	871.5073	871.5061	1.42	871.5010[M-H+HCOOH]$^-$;825.5464[M-H]$^-$; 783.4838[M-H-C2H2O]$^-$; 765.4728[M-H-C2H2O-H2O]$^-$; 621.4334[M-H-C2H2O-Glc]$^-$;459.3855[M-H-C2H2O-2Glc]$^-$

No	IDENTIFICATION	RT	FORMULE	M/Z	M/Z CAL	PPM	IONS
68	20(R)-ginsenoside Rs3	96.957	$C_{44}H_{74}O_{14}$	871.5067	871.5061	-0.73	871.5010[M-H+HCOOH]$^-$;825.5464[M-H]$^-$; 783.4838[M-H-C2H2O]$^-$; 765.4728[M-H-C2H2O-H2O]$^-$; 621.4334[M-H-C2H2O-Glc]$^-$;459.3855[M-H-C2H2O-3Glc]$^-$
69	Ginsenoside Rk1/Rg5	100.569	$C_{42}H_{70}O_{12}$	811.4871	811.4849	-2.67	811.4791[M-H+HCOOH]$^-$;765.4854[M-H]$^-$;603.4249[M-H-Glc]$^-$;441.3687[M-H-2Glc]$^-$
70	Ginsenoside Rk1/Rg6	101.639	$C_{42}H_{70}O_{13}$	811.4824	811.4849	3.11	811.4791[M-H+HCOOH]$^-$;765.4854[M-H]$^-$;603.4249[M-H-Glc]$^-$;441.3687[M-H-3Glc]$^-$

Table S2. LC–MS/MS data in the negative ion mode for common peaks identified in12 batches RGE

No	IDENTIFICATION	RT	FORMULE	M/Z	M/Z CAL	PPM	IONS
1	Notoginsenoside R3	28.337	$C_{48}H_{82}O_{19}$	1007.5424	1007.5358	2.77	1007.5375[M-H+HCOOH]$^-$;961.5355[M-H]$^-$;799.4811[M-Glc-H]$^-$; 637.4298[M-2Glc-H]$^-$； 475.3744[M-3Glc-H]$^-$
2	Re1/Re2/Re3/NG-N/VG-R4	32.472	$C_{48}H_{82}O_{19}$	1007.5421	1007.5407	1.73	1007.5421[M-H+HCOOH] $^-$;961.5372[M-H]$^-$; 799.4805[M-Glc-H]$^-$;637.4237[M-2Glc-H]$^-$; 475.3747[M-3Glc-H]$^-$
3	Notoginsenoside R1	33.208	$C_{47}H_{80}O_{18}$	977.5739	977.5327	3.47	977.5332[M-H+HCOOH]$^-$;931.5259[M-H]$^-$;799.4872[M-Arap-H]$^-$;637.4310 [M- Arap -Glc-H]$^-$
4	Ginsenoside Rg1	36.887	$C_{42}H_{72}O_{14}$	845.5235	845.5243	0.14	845.5235[M-H+HCOOH]$^-$;637.4520[M-H-Glc] $^-$;475.3940[M-H-2Glc]$^-$
5	Ginsenoside Re	36.564	$C_{48}H_{82}O_{18}$	991.5876	991.5893	1.99	991.5876[M-H+HCOOH]$^-$;945.5802[M-H]$^-$; 799.5148[M-H-Rha]$^-$; 637.4528[M-H--Rha Glc]$^-$; 475.3901[M-H-Rha-2Glc]$^-$
6	Notoginsenoside Rt	45.115	$C_{44}H_{74}O_{15}$	887.4717	887.4567	0.62	887.4717[M-H+HCOOH]$^-$;841.4797[M-H] $^-$; 637.4119[M-H-C_2H_2O-Glc]$^-$; 475.3662[M-H-C_2H_2O-2Glc]$^-$
7	Ginsenoside Ra3/R4	49.673	$C_{59}H_{10}0O_{27}$	1239.6907	1239.6902	0.5	1239.6907[M-H]$^-$;1077.6448[M-H-Xly]$^-$;945.5900[M-H-Xly-Glc]$^-$; 783.5144[M-H-Xly-2Glc]$^-$;621.4648[M-H-Xly-3Glc]$^-$;459.3907[M-H-Xly-4Glc]$^-$

No	IDENTIFICATION	RT	FORMULE	M/Z	M/Z CAL	PPM	IONS
8	Ginsenoside Rf	50.400	$C_{42}H_{72}O_{14}$	845.5204	845.4904	2.36	845.5204[M-H+HCOOH]$^-$;799.5129[M-H]$^-$;637.4518[M-H-Glc]$^-$; 475.3920[M-H-2Glc]$^-$;391.2939[M-H-2Glc-C6H12]$^-$;221.0694 1,3A2β； 101.0239 2,5A1β
12	Ginsenoside R4/Ra3	55.818	$C_{59}H_{100}O_{27}$	1239.6385	1239.6379	0.47	1239.6358[M-H]$^-$;1077.5830[M-H-Xly]$^-$;945.5326[M-H-Xly-Glc]$^-$; 783.4673[M-H-Xly-2Glc]$^-$;621.4356[M-H-Xly-3Glc]$^-$;459.3962[M-H-Xly-4Glc]$^-$
9	Ginsenoside Ra2/Ra1	54.814	$C_{58}H_{98}O_{26}$	1209.6777	1209.6732	2.02	915.5354[M-Glc-Xylp-H]$^-$;783.5187[M-Glc-Xylp-Araf-H]$^-$; 621.4573[M-2Glc-Xylp-Araf-H]$^-$;459.3967[M-3Glc-Xylp-Araf-H]$^-$; 459.3967[M-3Glc-Xylp-Araf-H]$^-$;
11	Notoginsenoside R2	55.483	$C_{41}H_{70}O_{13}$	815.5116	815.5110	0.68	815.5116[M-H+HCOOH]$^-$;769.5042[M-H];637.4514[M-H-Xylp]$^-$; 475.3929[M-H-Glc-Xylp]$^-$
10	Ginsenoside Rb1	55.023	$C_{54}H_{92}O_{23}$	1107.6414	1107.6426	0.75	1153.6413[M-H+HCOOH]$^-$;1107.6424[M-H]-;945.5788[M-H-Glc]$^-$; 783.5190[M-H-2Glc]$^-$;621.4607[M-H-3Glc]$^-$;459.4013[M-H-4Glc]$^-$
13	Ginsenoside Rg2	59.096	$C_{42}H_{72}O_{13}$	829.529	829.5278	3.65	829.5290[M-H+HCOOH]$^-$;783.5190[M-H]$^-$;637.4541[M-H-Rha]$^-$; 475.3945[M-H-Rha-Glc]$^-$;391.2955[M-H-Rha-Glc-C_6H_{12}]$^-$

No	IDENTIFICATION	RT	FORMULE	M/Z	M/Z CAL	PPM	IONS
14	Ginsenoside Rc	59.564	$C_{53}H_{90}O_{22}$	1077.6291	1077.6282	4.58	1077.6291[M-H]$^-$;945.5783[M-Araf-H]$^-$;783.5164[M-H-Araf-Glc]$^-$;621.4609[M-H-Araf-2Glc]$^-$;459.3980[M-H-Araf-3Glc]$^-$
15	Ginsenoside Ro	61.169	$C_{48}H_{76}O_{19}$	955.5309	955.5295	-2.72	955.5309[M-H]$^-$;793.4684 [M-H-Glc]$^-$;775.4506[M-H-H_2O-Glc]$^-$;731.4631[M-H-CO_2-H_2O-Glc]$^-$;613.3954[M-H-H2O-2Glc]$^-$;569.4022[M-H-CO2-H2O-2Glc]$^-$;455.3675[M-H-2Glc -GlcA]$^-$
16	Malonyl-ginsenoside Rb1	63.711	$C_{57}H_{94}O_{26}$	1193.5956	1193.5961	4.26	1193.5861[M-H]$^-$;1107.5948[M-H-Mal]$^-$;945.5394[M-H-Glc]$^-$;783.4839[M-H-2Glc]$^-$;621.4353[M-H-3Glc]$^-$;459.3817[M-H-4Glc]$^-$;323.0977[Glc-Glc-H]$^-$;179.0551[Glc-H]$^-$
17	Ginsenoside Rb2	64.915	$C_{53}H_{90}O_{22}$	1077.6311	1077.6314	1.26	1077.6311[M-H]$^-$;945.5896[M-Arap-H]$^-$;783.5236[M-Arap-Glc-H]$^-$;621.4609[M-Arap-2Glc-H]$^-$;459.4037[M-Arap-3Glc-H]$^-$
18	Ginsenoside Rb3	64.982	$C_{53}H_{90}O_{22}$	1077.6284	1077.6275	4.25	1077.6284[M-H]$^-$;945.5799[M-H-Xry]$^-$;783.5155[M-H-Xry-Glc]$^-$;621.4529[M-H-Xry-Glc]$^-$;293.0940[XryGlc-H]$^-$;149.0465[Xry-H]$^-$
19	Ginsenoside Rd	72.541	$C_{48}H_{82}O_{18}$	991.5879	991.5483	1.69	991.5879[M-H+HCOOH]$^-$;945.5791[M-H]$^-$;783.5167[M-H-Glc]$^-$;621.4575[M-H-2Glc]$^-$;459.3973[M-H-3Glc]$^-$;101.0238 2,5A1α/2,5A1β

No	IDENTIFICATION	RT	FORMULE	M/Z	M/Z CAL	PPM	IONS
20	Malonyl-Ginsenoside Rd	74.08	$C_{51}H_{84}O_{21}$	1031.5451	1031.5432	1.81	1031.5374[M-H]$^-$;945.5426[M-H-Mal]$^-$;783.4961[M-H-Glc-Mal]$^-$; 765.4784[M-H-H2O-Glc-Mal]$^-$;621.4375[M-H-2Glc-Mal]$^-$;459.3864[M-H-3Glc-Mal]$^-$
21	Ginsenoside Rs2/ Rs1	75.15	$C_{55}H_{92}O_{23}$	1165.6514	1165.6011	3.58	1165.5949[M-H+HCOOH]$^-$;1119.5988[M-H]$^-$;1077.5854[M-C_2H_2O-H]$^-$ 945.5429[M-C_2H_2O-Araf-H]$^-$;915.5755[M-C_2H_2O-Glc-H]$^-$;783.5176[M-C_2H_2O-Glc-Araf-H]$^-$; 621.4214[M-C_2H_2O-2Glc-Araf-H]$^-$;459.3903[M-C_2H_2O-3Glc-Araf-H]$^-$
22	Ginsenoside Rs2/ Rs1	75.752	$C_{55}H_{92}O_{23}$	1165.6022	1165.6011	4.67	1165.6514[M-H+HCOOH]$^-$;1119.6416[M-H]$^-$;1077.6260[M-C_2H_2O-H]$^-$; 945.5444[M-C_2H_2O-Arap-H]$^-$;915.5436[M-C_2H_2O-Glc-H]$^-$; 783.4980[M-C_2H_2O-Glc-Arap-H]$^-$;621.4163[M-C_2H_2O-2Glc-Arap-H]$^-$; 459.3801[M-C_2H_2O-3Glc-Arap-H]$^-$
23	Ginsenoside Rg9/Rg10	81.304	$C_{42}H_{70}O_{13}$	827.4785	827.4798	-2.14	827.4767[M-H+HCOOH]$^-$;781.4718[M-H]$^-$;619.4133[M-H-Glc]$^-$;457.3612[M-H-2Glc]$^-$
24	Ginsenoside Rg9/Rg10	79.966	$C_{42}H_{70}O_{13}$	827.4785	827.4798	-3.54	827.4767[M-H+HCOOH]$^-$;781.4759[M-H]$^-$;619.4167[M-H-Glc]$^-$; 457.3706[M-H-2Glc]$^-$
25	Notoginsenoside T5	82.976	$C_{41}H_{68}O_{12}$	797.4671	797.4693	2.65	797.4642[M-H+HCOOH]$^-$;751.4656[M-H]$^-$;619.4252[M-H-Xylp]$^-$; 457.3645[M-H-Xylp-Glc]$^-$

No	IDENTIFICATION	RT	FORMULE	M/Z	M/Z CAL	PPM	IONS
26	Ginsenoside Rg6/Rg4	84.047	$C_{42}H_{70}O_{12}$	811.4838	811.4849	2.88	811.4791[M-H+HCOOH]$^-$;765.4675[M-H]$^-$;619.4211[M-H-Rha]$^-$; 457.3609[M-H-Glc-Rha]$^-$
27	Ginsenoside Rg6/Rg4	85.251	$C_{42}H_{70}O_{12}$	811.4848	811.4849	3.11	811.4791[M-H+HCOOH]$^-$;765.4859[M-H]$^-$;619.4211[M-H-Rha]$^-$; 457.3609[M-H-Glc-Rha]$^-$
28	Chikusetsusaponin IVa	86.254	$C_{42}H_{66}O_{14}$	793.4369	793.4380	4.22	793.4329[M-H]$^-$;613.3735[M-Glc-H]$^-$;587.3984[M-Glc-CO2-H]$^-$; 455.3528[M-Glc-GlcA-H]$^-$
29	Ginsenoside Rh4/Rk3	87.257	$C_{36}H_{60}O_8$	665.4273	665.4270	-2.13	665.4235[M-H+HCOOH]$^-$;619.3950[M-H]$^-$;457.3587[M-H-Glc]$^-$
30	Ginsenoside Rh4/Rk4	87.194	$C_{36}H_{60}O_8$	665.4280	665.4270	3.65	665.4235[M-H+HCOOH]$^-$;619.3950[M-H]$^-$;457.3588[M-H-Glc]$^-$
31	20(S)-ginsenoside Rg3	89.665	$C_{42}H_{72}O_{13}$	829.4973	829.4955	4.25	829.4920[M-H+HCOOH]$^-$;783.4895[M-H]$^-$;621.4357[M-H-Glc]$^-$; 549.3828[M-H-2Glc]$^-$
32	20(R)-ginsenoside Rg3	90.535	$C_{42}H_{72}O_{13}$	829.4973	829.4955	-4.21	829.4920[M-H+HCOOH]$^-$;783.4897[M-H]$^-$;621.4385[M-H-Glc]$^-$; 459.3853[M-H-2Glc]$^-$
33	20(S)-ginsenoside Rs3	96.02	$C_{44}H_{74}O_{14}$	871.5073	871.5061	3.32	871.5010[M-H+HCOOH]$^-$;825.5464[M-H]$^-$;783.4838[M-H-C_2H_2O]$^-$; 765.4728[M-H-C_2H_2O-H_2O]$^-$;621.4334[M-H-C_2H_2O-Glc]$^-$;459.3855[M-H-C_2H_2O-2Glc]$^-$
34	20(R)-ginsenoside Rs3	96.957	$C_{44}H_{74}O_{14}$	871.5067	871.5061	2.58	871.5010[M-H+HCOOH]$^-$;825.5464[M-H]$^-$;783.4838[M-H-C_2H_2O]$^-$; 765.4728[M-H-C_2H_2O-H_2O]$^-$;621.4334[M-H-C2H2O-Glc]-;459.3855[M-H-C_2H_2O-3Glc]$^-$

No	IDENTIFICATION	RT	FORMULE	M/Z	M/Z CAL	PPM	IONS
35	Ginsenoside Rk1/Rg5	100.569	$C_{42}H_{70}O_{12}$	811.4871	811.4849	2.99	811.4791[M-H+HCOOH]$^-$;765.4854[M-H]$^-$;603.4249[M-H-Glc]$^-$;441.3687[M-H-2Glc]$^-$
36	Ginsenoside Rk1/Rg6	101.639	$C_{42}H_{70}O_{13}$	811.4871	811.4849	-3.32	811.4791[M-H+HCOOH]$^-$;765.4854[M-H]$^-$;603.4249[M-H-Glc]$^-$;441.3687[M-H-3Glc]$^-$

Table S3. Anti-Myocardial ischemia effect of 12 batches RGE (mean ±SD)

**Batch (RGE)**	**Cell viability**	**LDH activity**	**ROS level**	**ATP concentration**
**1**	0.772±0.063	0.756±0.073	0.505±0.056	0.646±0.268
**2**	0.669±0.063	0.887±0.126	0.422±0.177	0.569±0.084
**3**	0.518±0.039	0.695±0.142	0.497±0.117	0.266±0.071
**4**	0.769±0.067	0.823±0.115	0.473±0.088	0.345±0.071
**5**	0.789±0.030	0.791±0.087	0.439±0.076	0.417±0.056
**6**	0.710±0.077	0.762±0.152	0.483±0.141	0.516±0.071
**7**	0.685±0.159	0.658±0.126	0.317±0.286	0.576±0.178
**8**	0.463±0.076	0.258±0.138	0.209±0.354	0.852±0.115
**9**	0.631±0.076	0.347±0.279	0.449±0.152	0.654±0.087
**10**	0.553±0.126	0.545±0.220	0.357±0.084	0.603±0.156
**11**	0.667±0.034	0.004±0.272	0.289±0.232	0.762±0.089
**12**	0.667±0.046	0.127±0.315	0.269±0252	0.841±0.034

Table S4. LC–MS/MS data in the negative ion mode for identification of target cell extraction combined with cells

No	IDENTIFICATION	RT	FORMULE	M/Z	M/Z CAL	PPM	IONS
1	Ginsenoside Rg1	36.570	$C_{42}H_{72}O_{14}$	845.4925	845.4904	-2.47	845.4925[M-H+HCOOH]$^-$; 637.3475[M-H-Glc]$^-$; 475.3042[M-H-2Glc]$^-$
2	Ginsenoside Rf	52.166	$C_{42}H_{72}O_{14}$	845.4921	845.4904	-2	845.4957[M-H+HCOOH]$^-$;799.4869[M-H]$^-$; 637.4304[M-H-Glc]$^-$;475.3801[M-H-2Glc]$^-$
3	Notoginsenoside R2	55.030	$C_{41}H_{70}O_{13}$	815.4825	815.4978	-3.25	815.4825[M-H+HCOOH]$^-$;769.4743[M-H]; 637.3430[M-H-Xylp]$^-$; 475.4979[M-H-Glc-Xylp]$^-$
4	Ginsenoside Rb1	55.023	$C_{54}H_{92}O_{23}$	1153.6046	1153.6011	-2.99	1153.6046[M-H+HCOOH]$^-$; 1107.5987[M-H]$^-$; 945.5479[M-H-Glc]-;783.4832[M-H-2Glc]$^-$; 621.4303[M-H-3Glc]$^-$;459.9744[M-H-4Glc]$^-$
5	Ginsenoside Rg2	58.635	$C_{42}H_{72}O_{13}$	829.4994	829.4955	-4.7	829.4994[M-H+HCOOH]$^-$;783.4933[M-H]$^-$; 475.8112[M-H-Rha-Glc]$^-$; 391.9717[M-H-Rha-Glc-C6H12]$^-$
6	Ginsenoside Rb3	58.702	$C_{53}H_{90}O_{22}$	1123.5971	1123.5906	-5.8	1077.5891[M-H]$^-$;945.6899[M-Arap-H]$^-$; 783.4933[M-Arap-Glc-H]$^-$; 621.4260[M-Arap-2Glc-H]$^-$; 459.3817 [M-Arap-3Glc-H]$^-$
7	Ginsenoside Ro	61.664	$C_{48}H_{76}O_{19}$	955.4967	955.4908	-6.16	955.5309[M-H]-;793.4684 [M-H-Glc]$^-$; 775.4506[M-H-H2O-Glc]$^-$; 731.4477[M-H-CO_2-H2O-Glc]$^-$; 613.4180[M-H-H_2O-2Glc]$^-$; 569.3882[M-H-CO_2-H_2O-2Glc]$^-$; 455.3461 [M-H-2Glc -GlcA]$^-$

No	IDENTIFICATION	RT	FORMULE	M/Z	M/Z CAL	PPM	IONS
8	Ginsenoside Rb2	62.653	$C_{53}H_{90}O_{22}$	1123.5948	1123.5906	-3.75	1077.5916[M-H]$^-$; 945.5488[M-Arap-H]$^-$; 783.4918[M-Arap-Glc-H]$^-$; 621.4609[M-Arap-2Glc-H]$^-$;459.4037[M-Arap-3Glc-H]$^-$
9	Ginsenoside Rd	72.010	$C_{48}H_{82}O_{18}$	991.5515	991.5483	-3.21	991.5490[M-H+HCOOH]$^-$;945.5458[M-H]$^-$; 783.5001[M-H-Glc]$^-$; 621.4389[M-H-2Glc]$^-$; 459.1710[M-H-3Glc]$^-$
10	Ginsenoside Rg6/Rg4	83.714	$C_{42}H_{70}O_{12}$	811.4857	811.4849	-0.95	811.4857[M-H+HCOOH]$^-$;765.4814[M-H]$^-$;619.4175[M-H-Rha]$^-$; 457.3781[M-H-Glc-Rha]$^-$
11	Ginsenoside Rg6/Rg4	84.974	$C_{42}H_{70}O_{12}$	811.4897	811.4849	-5.87	811.4758[M-H+HCOOH]$^-$;765.4839[M-H]$^-$;619.4089[M-H-Rha]$^-$; 457.3638[M-H-Glc-Rha]$^-$
12	Ginsenoside Rh4/Rk3	86.046	$C_{36}H_{60}O_8$	665.4294	665.4270	-3.57	665.4299[M-H+HCOOH]$^-$;619.4241[M-H]$^-$;457.3665[M-H-Glc]$^-$
13	Ginsenoside Rh4/Rk4	87.587	$C_{36}H_{60}O_8$	665.4313	665.4270	-6.42	665.4283[M-H+HCOOH]$^-$;619.4232[M-H]$^-$;457.4178[M-H-Glc]$^-$
14	20(S)-ginsenoside Rg3	89.370	$C_{42}H_{72}O_{13}$	829.4985	829.4955	-3.62	829.4993[M-H+HCOOH]$^-$;783.4921[M-H]$^-$;621.4371[M-H-Glc]$^-$; 459.3733[M-H-2Glc]$^-$
15	20(R)-ginsenoside Rg3	90.217	$C_{42}H_{72}O_{13}$	829.4995	829.4955	-4.46	829.4982 [M-H+HCOOH]$^-$;783.4825[M-H]$^-$;621.4304[M-H-Glc]$^-$; 459.2037[M-H-2Glc]$^-$

No	IDENTIFICATION	RT	FORMULE	M/Z	M/Z CAL	PPM	IONS
16	Ginsenoside Rk1/Rg5	100.267	$C_{42}H_{70}O_{12}$	811.4877	811.4849	-3.41	811.4861[M-H+HCOOH]$^-$; 765.4803[M-H]-;603.4233[M-H-Glc]$^-$; 441.9177[M-H-2Glc]$^-$
17	Ginsenoside Rk1/Rg5	101.471	$C_{42}H_{70}O_{13}$	811.4856	811.4849	-0.82	811.4844[M-H+HCOOH]$^-$;765.4806[M-H]$^-$;603.4179[M-H-Glc]$^-$; 441.3294[M-H-3Glc]$^-$

Table S5. The result of GRA model and PLS model.

	GRA (Correlation coefficient)				PLS (VIP value)			
	MTT-GRA	LDH-GRA	ATP-GRA	ROS-GRA	MTT-PLS	LDH-PLS	ATP-PLS	ROS-PLS
PEAK1	1.095564	1.042833	1.091511	1.129462	1.138134	0.969394	0.811433	0.924449
PEAK2	1.021934	1.034972	0.997137	1.07672	1.288984	0.955944	1.407459	0.833158
PEAK3	1.027012	0.99698	1.030665	1.074147	1.477286	0.935253	0.933775	0.909759
PEAK4	1.052401	1.066414	1.009555	1.102448	0.904057	0.757307	0.784245	0.763903
PEAK5	1.0067	1.029732	0.994654	0.980239	0.570108	1.072851	0.765423	0.760756
PEAK6	1.002892	1.048073	0.984719	1.022691	1.070512	0.674541	0.532241	1.526758
PEAK7	0.968616	0.961607	0.961126	0.995676	1.249451	0.824554	0.763332	0.732424
PEAK8	1.018125	1.023181	0.974785	1.067715	1.138134	0.811104	0.766469	0.826863
PEAK9	1.05621	1.069035	0.984719	1.047132	1.316033	0.782136	1.015336	1.263379
PEAK10	0.964807	0.970778	0.922631	1.003395	1.249451	0.93008	1.081213	0.700944
PEAK11	0.943226	0.981258	0.935049	0.927497	0.533696	0.732477	0.779017	0.790136
PEAK12	0.985119	1.00484	0.972302	0.953225	0.585713	1.109061	0.69641	0.798531
PEAK13	1.082869	1.045453	1.117588	1.050992	1.412785	1.083197	1.309167	1.532004
PEAK14	0.920375	0.974708	0.891587	0.937788	0.72928	0.731442	0.730917	0.730325
PEAK15	1.066366	1.053313	1.125039	1.034268	1.121489	1.119407	1.308121	1.352571
PEAK16	0.848015	0.903963	0.853092	0.837449	0.655416	0.67661	0.707912	0.762854
PEAK17	0.88483	0.86335	0.889104	0.886332	0.642931	1.204242	1.169048	0.752361

PEAK18	0.88356	0.920994	0.892829	0.859317	0.543059	0.662126	0.776926	0.796432
PEAK19	1.03209	1.084756	1.030665	1.034268	1.12565	1.607725	1.562217	1.326338
PEAK20	1.033359	0.940646	1.113863	0.991817	1.284823	1.94603	1.991983	2.181532
PEAK21	0.978771	0.981258	0.976027	0.958371	0.506647	0.894905	0.611711	0.83001
PEAK22	0.85944	0.906583	0.867994	0.841308	0.715755	0.939391	0.708958	0.84575
PEAK23	0.869596	0.941956	0.892829	0.873468	0.81979	0.974567	0.816661	0.802728
PEAK24	1.068905	0.991739	1.089028	1.07672	1.309791	1.415294	1.423144	1.162644
PEAK25	0.821356	0.889552	0.818323	0.851599	1.502254	0.920769	1.117811	0.92235
PEAK26	1.034629	1.054624	1.014522	1.014972	0.683505	0.800759	0.985012	0.976915
PEAK27	1.040976	1.012701	1.015764	1.07286	1.049705	0.934218	0.817707	0.757608
PEAK28	0.892447	0.941956	0.927598	0.867036	1.076754	0.944564	0.929592	0.972718
PEAK29	1.028281	0.987809	1.061709	1.012399	0.874928	1.174239	0.8794	0.826863
PEAK30	1.046054	1.019251	1.017005	1.080579	1.036181	0.880421	0.792611	0.762854
PEAK31	1.063827	1.033662	1.059225	1.02655	0.845798	1.007673	1.130359	1.142707
PEAK32	1.067635	1.037592	1.080335	1.035555	0.891573	1.122511	1.230742	1.218258
PEAK33	1.075252	1.045453	1.09772	1.056137	1.047625	1.168032	1.247473	1.177335
PEAK34	1.085408	1.052003	1.098962	1.106307	1.324356	0.787309	0.926455	0.700944
PEAK35	1.099372	1.048073	1.127522	1.075433	1.364929	1.337702	1.262112	1.429171
PEAK36	1.073983	1.038902	1.082819	1.038128	0.91342	1.112165	1.227605	1.205666
